# Importance of urgent surgical treatment in a patient having massive pericardial effusion with end-stage renal disease

**DOI:** 10.1186/1749-8090-8-S1-P60

**Published:** 2013-09-11

**Authors:** K Ergunes, H Iner, E Celik, U Yetkin, A Gurbuz

**Affiliations:** 1 Izmir Katip Celebi Üniversity Ataturk Training and Research Hospital, Department of Cardiovascular Surgery, Izmir, Turkey

## Background

Massive pericardial effusion in patients with end-stage renal disease is potentially life-threatening condition.

## Method

A 79-year male with end-stage renal disease who was maintenance dialysis was admitted to our hospital. He had chest pain, dyspnea, and weakness. The diagnosis of pericardial effusion was confirmed by echocardiogram.

## Results

Subxiphoid pericardial drainage was performed under local anesthesia. Pericardial fluid of 1000 ml was drained. A pericardial drainage tube was inserted at surgery and removed after 5 days. A 2x3 cm2 biopsy specimen was taken under direct vision from the lower aspect of anterior pericardium. Sample of the drained fluid was collected for microbiological culture and cytological analysis. Microorganism cultured no from pericardial fluid. The pericardial biopsy specimen was negative for malignancy. He has uremic pericardial effusion. There was no complication and surgery-related death. After hospital discharge, patient was followed with physical examinations and echocardiogram.

## Conclusions

We advise a subxiphoid pericardial window with pericardial drainage under local anesthesia for and-stage renal disease patients on dialysis having a symptomatic and massive pericardial effusion.

